# New nomogram for predicting lymph node positivity in pancreatic head cancer

**DOI:** 10.3389/fonc.2023.1053375

**Published:** 2023-01-25

**Authors:** Xingren Guo, Xiangyang Song, Xiaoyin Long, Yahui Liu, Yixin Xie, Cheng Xie, Bai Ji

**Affiliations:** The Department of General Surgery Center-Hepatobiliary and Pancreatic Surgery, The First Affiliated Hospital of Jilin University, Changchun, China

**Keywords:** LNM, nomogram, pancreatic head cancer, clinical indicators, age

## Abstract

**Background:**

Pancreatic cancer is one of the most malignant cancers worldwide, and it mostly occurs in the head of the pancreas. Existing laparoscopic pancreaticoduodenectomy (LPD) surgical techniques have has undergone a learning curve, a wide variety of approaches for the treatment of pancreatic cancer have been proposed, and the operation has matured. At present, pancreatic head cancer has been gradually changing from “surgeons’ evaluation of anatomical resection” to “biologically inappropriate resection”. In this study, the risk of lymph node metastasis in pancreatic head cancer was predicted using common preoperative clinical indicators.

**Methods:**

The preoperative clinical data of 191 patients with pancreatic head cancer who received LPD in the First Affiliated Hospital of Jilin University from May 2016 to December 2021 were obtained. A univariate regression analysis study was conducted, and the indicators with a significance level of P<0.05 were included in the univariate logistic regression analysis into multivariate. Lastly, a nomogram was built based on age, tumor size, leucocyte,albumin(ALB), and lymphocytes/monocytes(LMR). The model with the highest resolution was selected by obtaining the area under a curve. The clinical net benefit of the prediction model was examined using decision curve analyses.Risk stratification was performed by combining preoperative CT scan with existing models.

**Results:**

Multivariate logistic regression analysis found age, tumor size, WBC, ALB, and LMR as five independent factors. A nomogram model was constructed based on the above indicators. The model was calibrated by validating the calibration curve within 1000 bootstrap resamples. The ROC curve achieved an AUC of 0.745(confidence interval of 95%: 0.673-0.816), thus indicating that the model had excellent discriminative skills. DCA suggested that the predictive model achieved a high net benefit in the nearly entire threshold probability range.

**Conclusions:**

This study has been the first to investigate a nomogram for preoperative prediction of lymphatic metastasis in pancreatic head cancer. The result suggests that age, ALB, tumor size, WBC, and LMR are independent risk factors for lymph node metastasis in pancreatic head cancer. This study may provide a novel perspective for the selection of appropriate continuous treatment regimens, the increase of the survival rate of patients with pancreatic head cancer, and the selection of appropriate neoadjuvant therapy patients.

## Introduction

Pancreatic cancer is one of the most malignant tumors worldwide, the five-year survival rate is less than 5%, and 75% occurs in the pancreatic head (1). When patients are diagnosed with pancreatic cancer, most have lost the opportunity for surgery. Feasible pancreaticoduodenectomy without distant metastasis.

Pancreaticoduodenectomy has been confirmed as one of the largest operations in general surgery. LPD has experienced a learning curve in many large tertiary hospitals with shorter postoperative recovery times and fewer complications over the past few years. However, a considerable amount of research has suggested that the survival rate of postoperative patients remains not ideal (2, 3). On the one hand, it is dependent on the malignant biological characteristics of pancreatic cancer. On the other hand, numerous patients have lymph node metastasis before surgery (4), resulting in poor surgical results. Existing research has shown that preoperative lymph node metastasis is an independent risk factor for the postoperative survival rate of patients. The guidelines also recommend preoperative neoadjuvant therapy for patients with positive large regional lymph nodes (5).

Accordingly, preoperative prediction of lymph node metastasis takes on a critical significance to neoadjuvant therapy. At present, it is still difficult to predict lymph node metastasis by preoperative imaging indicators (6). Currently, a number of imaging modalities, such as endoscopic ultrasonography (EUS), computed tomography (CT), magnetic resonance imagin (MRI), and Positron emission tomography (PET), have been used to identify lymph node metastases (LNM). Radiologists often judge LNM by the size of the lymph node, the smooth edge of the lymph node and the homogeneous density or signal on CT or MRI images. Positron emission tomography/computed tomography (PET/CT), in addition to offering anatomical information, can provide an intuitive picture of the metabolic status of the lesion through semi-quantitative parameters such as standard uptake values and total glycolysis of the lesion. For instance, if the lymph node is present in a high uptake state on a PET image, it is highly suspected to be malignant. However, its accuracy in predicting lymph node metastasis in patients with pancreatic cancer is not very high (7–10). In recent years, Serum markers MMP7, MUC1, and MUC2 have been used to detect the preoperative status of PDAC lymph nodes and the rise of radiology, but their clinical application has been limited due to technical restriction and low accuracy (11, 12). Because, we use the advantage of nomogram, combined with preoperative clinical common indicators to predict the probability of lymph node cancer of the head of the pancreas.

In this study, there were 129 patients with positive lymph nodes, and only 34 patients had lymph node metastasis confirmed by preoperative imaging, and the predictive rate only reached 26%. This study aimed to investigate the correlation between common preoperative clinical indicators and lymph node metastasis(LNM) of pancreatic head cancer and to construct a corresponding nomogram to better identify patients with positive lymph nodes, which has potential significance for individualized comprehensive treatment.

## Materials and procedures

### Patients

Evaluation of the therapeutic information of patients with pancreatic head cancer who had LPD between May 2016 to December 2021 at the First Affiliated Hospital of Jilin University. This study was done in line with the Helsinki Declaration, with the agreement of the Research Ethics Committee of the First Affiliated Hospital of Jilin University, and with the informed consent of all patients.

The inclusion criteria are presented as follows:

(1) The pathological results were pancreatic carcinoma; (2) The lesion was located in the head of the pancreas; (3) Thin-layer CECT was performed in 191 patients within 1 month before operation and (4) There was a minimum number of LNs (eln) of 12 examinations.

The exclusion criteria are presented as follows:

(1) distant metastases (liver metastases or peritoneal carcinomatosis) on surgical exploration (2) preoperative anticancer therapy (chemotherapy, radiotherapy, or both) (3) incomplete clinicopathological data.

### Establishment of cutoff values for variables and pathological characteristics

We recorded the LNM indices of Pancreatic head cancer based on the postoperative pathological report after analyzing the routine and preoperative blood biochemical test findings. The ideal cutoff values for the variables in this study were established using receiver operating characteristic curves and the maximum Youden index. The definition of LSR was ALT (U/L)/AST (U/L). LMR was determined as lymphocytes (109/L)/monocytes (109/L). Their cutoff levels were set based on the receiver operating characteristic curve and the highest Youden index. At P less than 0.05, differences achieved statistical significance. Lastly, 191 patients were included, of which 129 patients had lymph node metastases and 62 patients had no lymph node metastases. The information regarding patients is listed in **Table 1**.

**Table 1:** Patients Characteristics.

Variables, n (%)	Level	Total (n=191)	ILM negative (n=62)	ILM positive (n=129)	p	
Gender	0	82 (42.932)	32 (51.613)	50 (38.760)	0.093	Chi-square test
	1	109 (57.068)	30 (48.387)	79 (61.240)		
Age	0	99 (51.832)	25 (40.323)	74 (57.364)	0.027	Chi-square test
	1	92 (48.168)	37 (59.677)	55 (42.636)		
Tumorsize	0	73 (38.220)	31 (50.000)	42 (32.558)	0.02	Chi-square test
	1	118 (61.780)	31 (50.000)	87 (67.442)		
CA125	0	80 (41.885)	31 (50.000)	49 (37.984)	0.115	Chi-square test
	1	111 (58.115)	31 (50.000)	80 (62.016)		
CA199	0	104 (54.450)	40 (64.516)	64 (49.612)	0.053	Chi-square test
	1	87 (45.550)	22 (35.484)	65 (50.388)		
ALP	0	56 (29.319)	28 (45.161)	28 (21.705)	<0.001	Chi-square test
	1	135 (70.681)	34 (54.839)	101 (78.295)		
ALB	0	118 (61.780)	30 (48.387)	88 (68.217)	0.008	Chi-square test
	1	73 (38.220)	32 (51.613)	41 (31.783)		
GLOB	0	150 (78.534)	42 (67.742)	108 (83.721)	0.012	Chi-square test
	1	41 (21.466)	20 (32.258)	21 (16.279)		
DBIL	0	68 (35.602)	30 (48.387)	38 (29.457)	0.011	Chi-square test
	1	123 (64.398)	32 (51.613)	91 (70.543)		
IBIL	0	36 (18.848)	19 (30.645)	17 (13.178)	0.004	Chi-square test
	1	155 (81.152)	43 (69.355)	112 (86.822)		
WBC	0	133 (69.634)	51 (82.258)	82 (63.566)	0.009	Chi-square test
	1	58 (30.366)	11 (17.742)	47 (36.434)		
NEU	0	128 (67.016)	48 (77.419)	80 (62.016)	0.034	Chi-square test
	1	63 (32.984)	14 (22.581)	49 (37.984)		
PCT	0	171 (89.529)	59 (95.161)	112 (86.822)	0.078	Chi-square test
	1	20 (10.471)	3 (4.839)	17 (13.178)		
MPV	0	64 (33.508)	25 (40.323)	39 (30.233)	0.167	Chi-square test
	1	127 (66.492)	37 (59.677)	90 (69.767)		
PDW	0	79 (41.361)	31 (50.000)	48 (37.209)	0.093	Chi-square test
	1	112 (58.639)	31 (50.000)	81 (62.791)		
TT	0	34 (17.801)	8 (12.903)	26 (20.155)	0.22	Chi-square test
	1	157 (82.199)	54 (87.097)	103 (79.845)		
APTT	0	59 (30.890)	24 (38.710)	35 (27.132)	0.105	Chi-square test
	1	132 (69.110)	38 (61.290)	94 (72.868)		
PT	0	92 (48.168)	26 (41.935)	66 (51.163)	0.232	Chi-square test
	1	99 (51.832)	36 (58.065)	63 (48.837)		
INR	0	129 (67.539)	38 (61.290)	91 (70.543)	0.201	Chi-square test
	1	62 (32.461)	24 (38.710)	38 (29.457)		
FBG	0	61 (31.937)	16 (25.806)	45 (34.884)	0.208	Chi-square test
	1	130 (68.063)	46 (74.194)	84 (65.116)		
LMR	0	181 (94.764)	54 (87.097)	127 (98.450)	<0.001	Chi-square test
	1	10 (5.236)	8 (12.903)	2 (1.550)		
LSR	0	48 (25.131)	24 (38.710)	24 (18.605)	0.003	Chi-square test
	1	143 (74.869)	38 (61.290)	105 (81.395)		

### Statistical analyses

**Figure 1:**
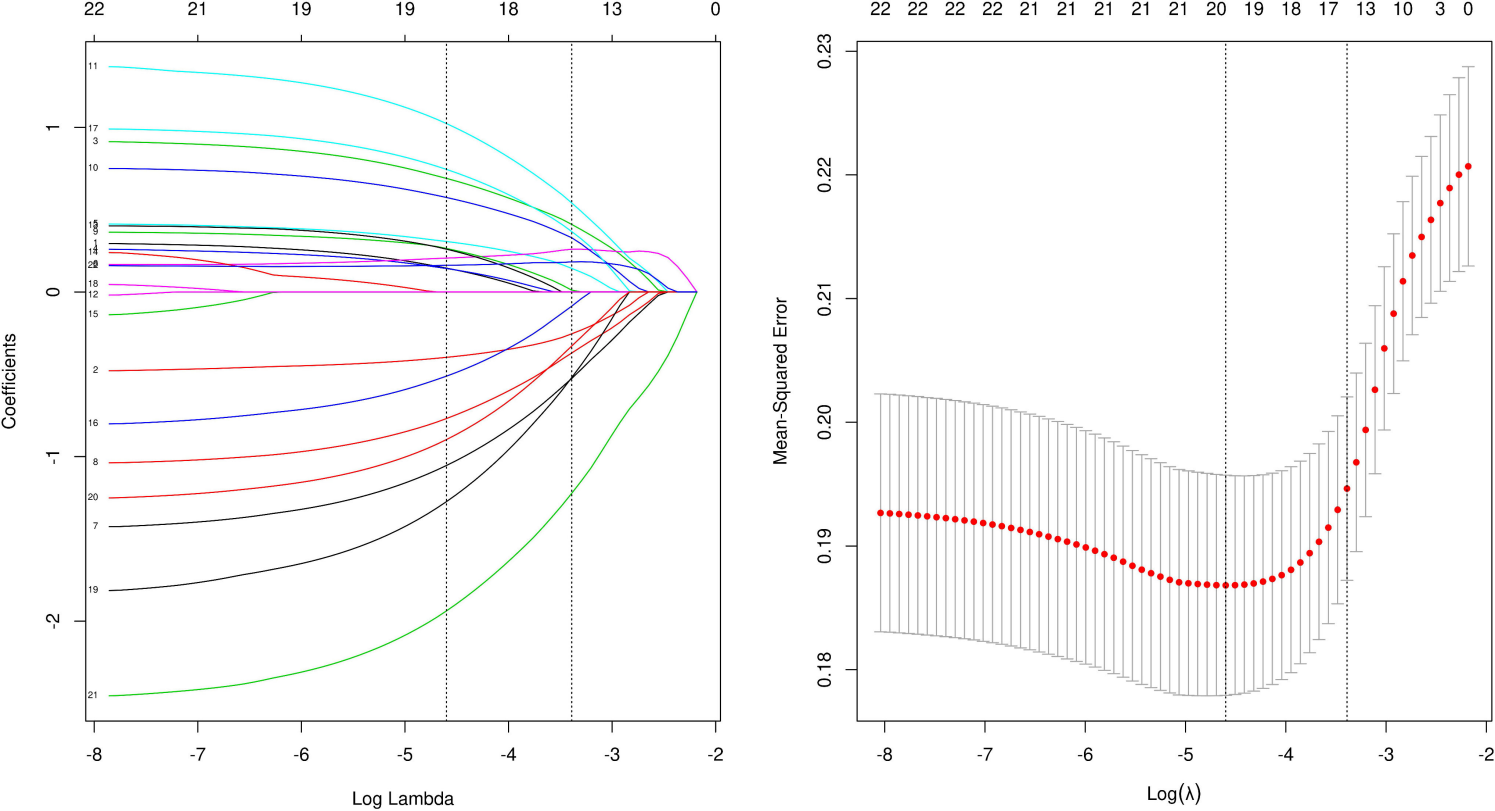
lasso analysis.

Cutoffs were determined by transforming continuous information into categorical variables based on the ROC’s maximum Youden index (sensitivity plus specificity minus 1). Categorical variables are described as numbers (percentages). LASSO regression analysis was used for data dimensionality reduction and element selection. (**Figure 1**) Between-group heterogeneity was compared through the chi-square test. Using univariate and multivariate logistic regression analysis, odds ratios (ORs) and 95% confidence intervals (CIs) were generated, of which OR>1 results indicated that the variable was a risk factor. Differences achieved statistical significance if P was less than 0.05. In the final nomogram model, the indicators with P less than 0.05 were included into the multiple logistic regression, and the nomogram model was built. The ROC of the model was obtained to evaluate its performance, a thousand bootstrapping was performed, a calibration curve was generated, and then a DCA curve was generated to evaluate the net benefit of the model. (R4.1.2 and SPSS26.0 were used for data processing and statistical analysis)Finally, risk stratification was performed by combining preoperative CT and existing models.

## Results

### Fundamental attributes and limit values of the variables

191 patients who had LPD between May 2016 to December 2021 at the First Affiliated Hospital of Jilin University were included, with 109 males and 82 females. **Table 1** lists the clinical features of the patients. The size of the tumor was extracted from preoperative CT-enhanced scan reports (e.g., imaging data). 129 out of 191 patients had positive lymph node, whereas 62 did not.

### Independent preoperative risk factors for LNM

In the univariate logistic regression evaluation, age less than 61 years old, tumor size was equals to or over 2.6cm, Alkaline phosphatase(ALP) was equals to or more than 186U/L, ALB was less than 40.5g/L, globulin was less than 30.1g/L, Direct Bilirubin(DBIL) was equals to or more than52.8μmol/L,Indirect bilirubin (IBIL) was equals to or more than12.5μmol/L, WBC was equals to or more than 6.71 10^9/L, neutrophil (NEU) was equals to or more than4.19 10^9/L, LMR was less than 8.125, and LSR was equals to or more than 1.1. After multivariate logistic regression analysis, only tumor size was equals to or more than 2.6cm (odds ratio [OR] was equals to 2.259, 95% CI: 1.126-4.598, P was equals to 0.023), ALB was less than 40.5g/L(odds ratio [OR] equals to 0.429, 95% CI: 0.203-0.893, P was equals to 0.024), LMR was less than 8.125(odds ratio [OR] was equals to 0.169, 95% CI: 0.022-0.831, P was equals to 0.044), which were the preoperative independent risk factors for LNM in patients with pancreatic head cancer. **Tables 2** and **3** list the results of univariate and multivariate regression analysis.

**Table 2:** Univariate regression analysis.

Variables	N	OR	95%CI	p	auc	cutoff
Gender						
0	82				0.564	1
1	109	1.685	[0.915,3.105]	0.094		
Age						
0	99				0.562	61
1	92	0.502	[0.271,0.930]	0.028		
Tumorsize						
0	73				0.565	2.6
1	118	2.071	[1.115,3.848]	0.021		
CA125						
0	80				0.538	11.57
1	111	1.633	[0.886,3.010]	0.116		
CA199						
0	104				0.548	219.33
1	87	1.847	[0.989,3.448]	0.054		
ALP						
0	56				0.594	186
1	135	2.971	[1.547,5.703]	0.001		
ALB						
0	118				0.601	40.5
1	73	0.437	[0.235,0.813]	0.009		
GLOB						
0	150				0.569	30.1
1	41	0.408	[0.201,0.829]	0.013		
DBIL						
0	68				0.6	52.8
1	123	2.245	[1.201,4.197]	0.011		
IBIL						
0	36				0.58	12.5
1	155	2.911	[1.385,6.119]	0.005		
WBC						
0	133				0.547	6.71
1	58	2.657	[1.263,5.591]	0.01		
NEU						
0	128				0.532	4.19
1	63	2.1	[1.050,4.201]	0.036		
PCT						
0	171				0.506	0.37
1	20	2.985	[0.841,10.600]	0.091		
MPV						
0	64				0.514	10.9
1	127	1.559	[0.829,2.932]	0.168		
PDW						
0	79				0.522	13.3
1	112	1.687	[0.915,3.114]	0.094		
TT						
0	34				0.515	13.4
1	157	0.587	[0.249,1.384]	0.224		
APTT						
0	59				0.512	27.5
1	132	1.696	[0.893,3.222]	0.107		
PT						
0	92				0.53	11.3
1	99	0.689	[0.374,1.270]	0.233		
INR						
0	129				0.521	1.02
1	62	0.661	[0.350,1.249]	0.202		
FBG						
0	61				0.513	3.36
1	130	0.649	[0.331,1.274]	0.209		
LMR						
0	181				0.523	8.125
1	10	0.106	[0.022,0.517]	0.005		
LSR						
0	48				0.597	1.118787879
1	143	2.763	[1.405,5.436]	0.003		

**Table 3:** Multivariate regression analysis.

Variables	OR	Lower	Upper	p
Age	0.516	0.247	1.055	0.072
Tumorsize	2.259	1.126	4.598	0.023
ALP	1.224	0.411	3.485	0.709
ALB	0.429	0.203	0.893	0.024
GLOB	0.532	0.239	1.196	0.123
DBIL	0.974	0.336	2.632	0.959
IBIL	1.868	0.535	6.686	0.328
WBC	2.22	1.009	5.201	0.055
LMR	0.169	0.022	0.831	0.044
LSR	1.368	0.539	3.4	0.502

### Development and validation of the novel preoperative LNM prediction nomogram

**Figure 2:**
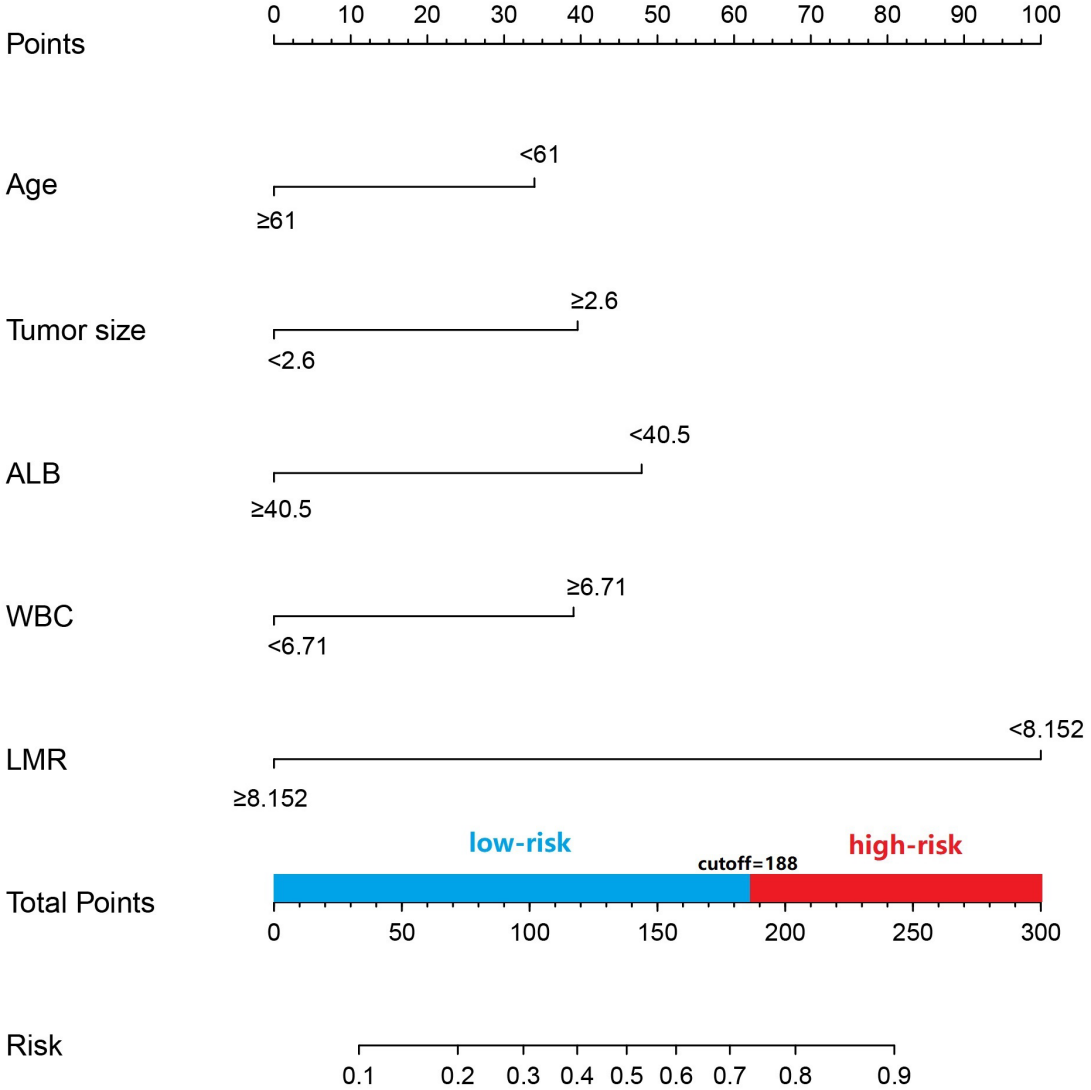
Prediction of LNM in patients with pancreatic head cncer using a nomogram. To get the position of each factor on the corresponding axis, draw lines on the point axis to represent the number of points. Add all the scores and find the place of the total score to determine the probability of LNM for that line in the nomogram.

**Figure 3:**
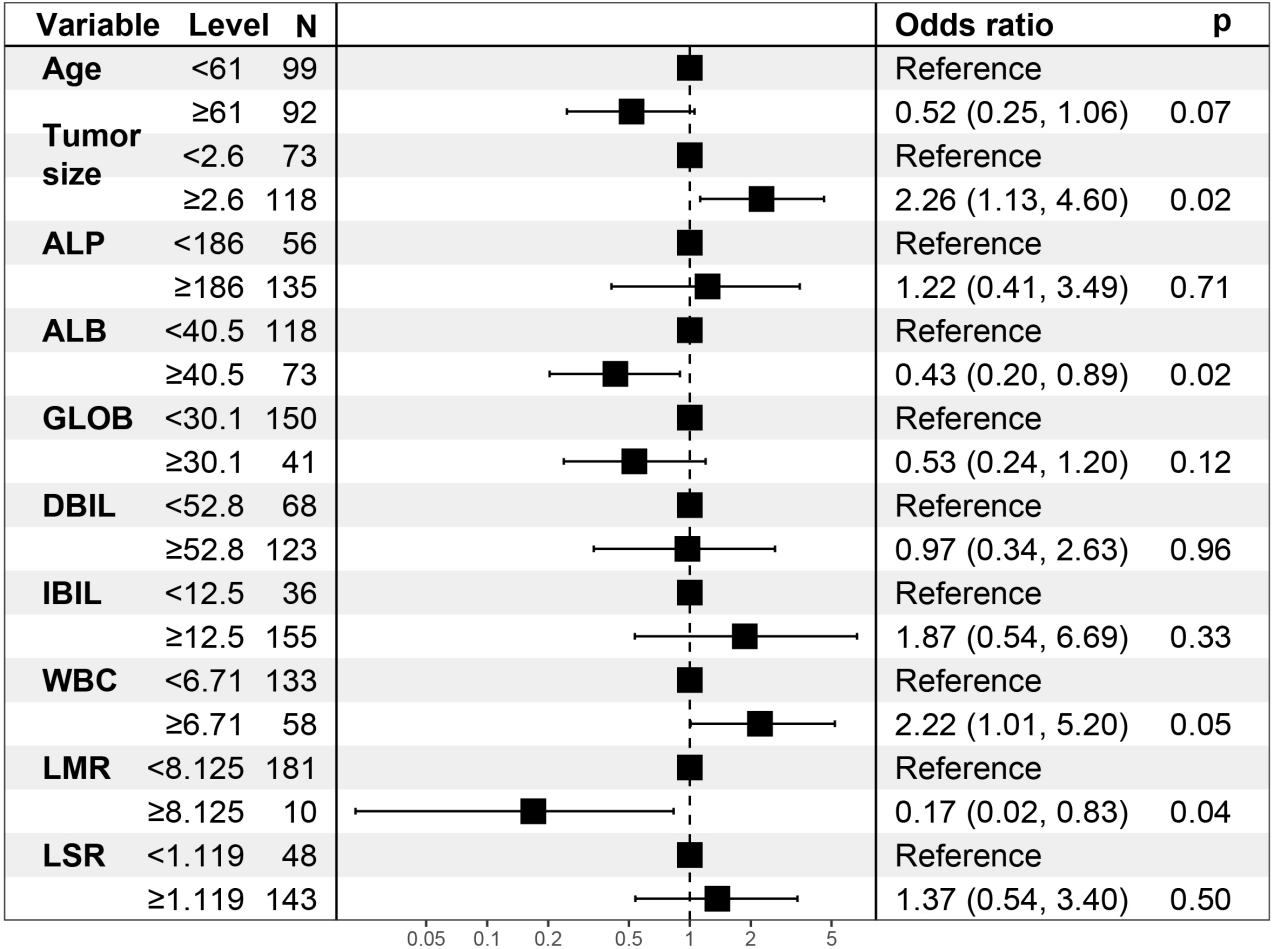
Model’s forest plot.

**Figure 4:**
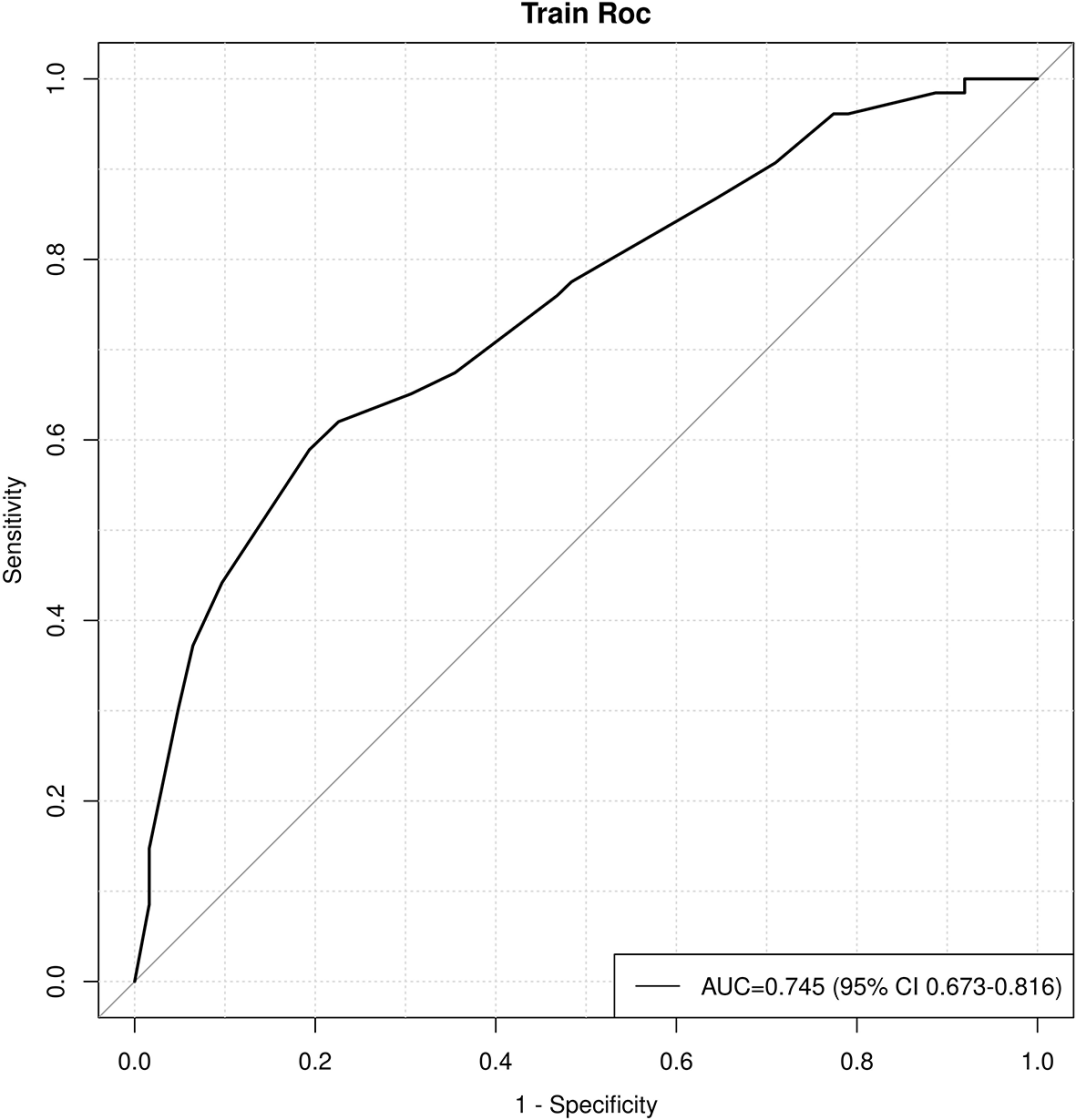
The ROC curve of the model.

**Figure 5:**
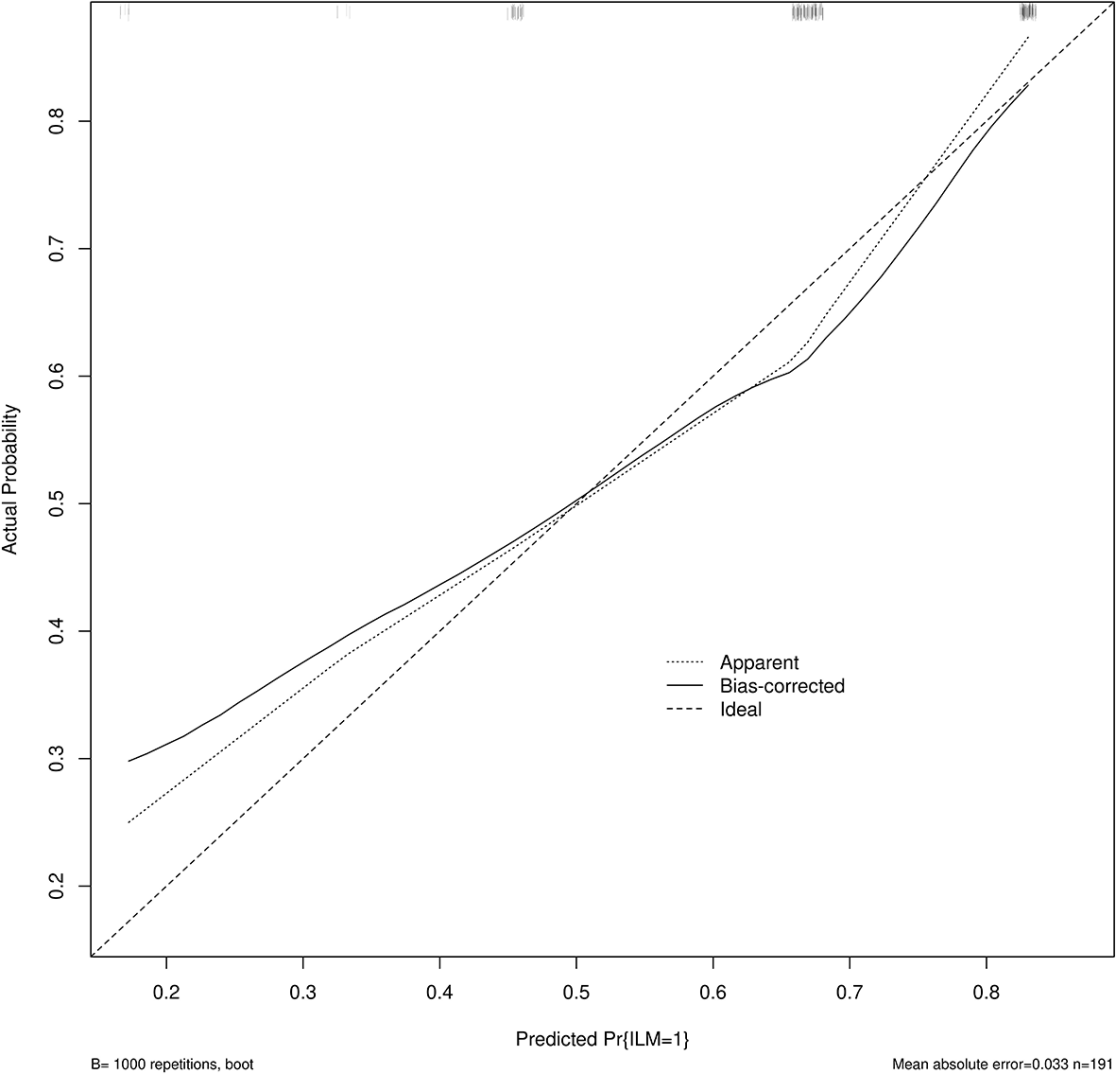
Calibration curve for predicting LNM. The nomogram predicted LNM is plotted on the x-axis, and the actual incidence of LNM is plotted on the y-axis. A plot along the 45° line will indicate a perfectly calibrated model where the predicted LNM is the same as the actual LNM. The expected probability distribution for the occurrence of LNM is shown at the top of the figure.

**Figure 6:**
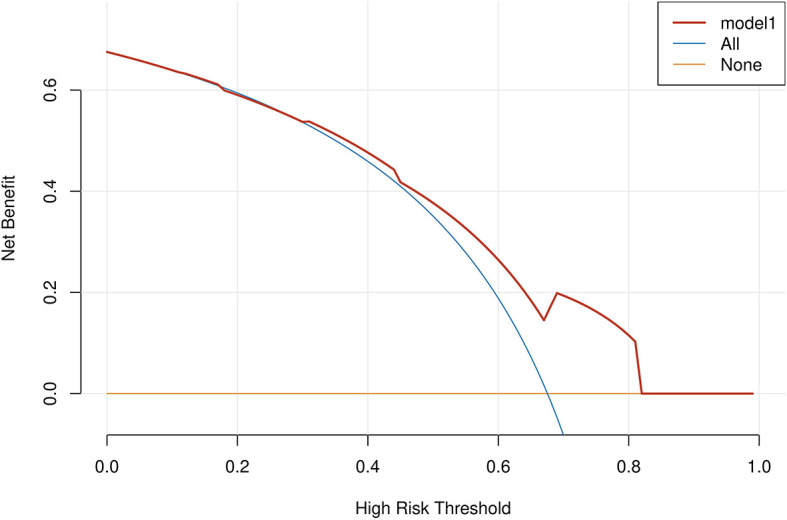
A decision curve analysis (DCA) was performed on the nomogram of the model. The solid black line assumes that all patients are LNM positive or negative, respectively. The dashed lines represent the net payoff of the nomogram at different threshold probabilities.

Age, tumor size, WBC, LMR, and ALB were taken based on the multiple logistic regression analysis of the training group to generate a nomogram and Forest plot to predict LNM in patients with pancreatic head cancer before surgery (**Figures 2**, **3**). The total score of the integral nomogram formula may be obtained by adding the scores for the respective element, and the probability of MVI can be predicted based on the sum of the integrals. Under the total score was higher than 188 points, it is considered a high risk of lymph node metastasis. Under the total score of less than 188 points, it was considered a low risk of lymph node metastasis. The nomogram prediction model achieved a high degree of predictive capacity, as indicated by the result. The AUC area for this model was 0.745 (**Figure 4**). (95% CI 0.673-0.816). The result of the model indicated that the standard curve was well consistent with the predicted curve, thus suggesting agreement between the observed frequencies and projected probability of MVI (**Figure 5**). The result of DCA indicated that the predictive model had a high net benefit throughout almost the entire threshold probability range, thus suggesting that the new nomogram had considerable clinical use (**Figure 6**).

### The establishment of risk stratification model

Risk stratification was performed on the existing model with a low risk score of 0, a high risk score of 1, and a preoperative CT lymph node positive score of 1 and a negative score of 0, using a combination of preoperative CT examination and the model, if the cumulative score≥1, the high-risk group, the cumulative score 0 for low-risk group, the final model has statistical significance. (p <0.05)

## Discussion

Pancreatic cancer is the “king of cancers” and overall survival rates for patients with ductal adenocarcinoma of the pancreas have barely improved over the last few decades (13–16). Statistics published in the United States (17) over the past few years have suggested that it is the fourth leading cause of death from cancer, one of the main reasons being its susceptibility to early metastasis through lymphatic drainage, and many studies have confirmed the adverse effects of lymphatic metastasis (18, 19). Thus, the stage of pancreatic cancer should be correctly evaluated, and lymph node status should be accurately reported, which is also conducive to evaluating the prognostic status of the patient and determining the best treatment plan. Through the analysis of the potential factors promoting lymph node metastasis (LNM), several positive indicators were identified, which can be evaluated preoperatively in conjunction with the American Joint Committee on Cancer (AJCC) 8th edition staging system for ductal adenocarcinoma of the pancreas (17) to determine whether a patient can undergo surgery or radiotherapy to achieve the optimal prognosis.

Studies have shown that neoadjuvant therapy enables a better survival rate than surgery alone (20), that lymph node positivity is a risk factor for poor outcomes in postoperative patients (20, 21), and that postoperative chemotherapy for patients with lymph node positivity has been shown to improve median survival and survival after surgery (22). In recent years, with the maturity of surgical techniques, postoperative chemotherapy has gradually achieved some results. Preoperative neoadjuvant chemotherapy has become a research hotspot for pancreatic cancer. This study is the first convincing demonstration of clear benefits of preoperative neoadjuvant therapy in node-positive patients (23), on the one hand, achieving reduced nodal staging and thus improved patient survival, and on the other hand, during this period of neoadjuvant therapy, patients with high-risk biological behavioral violations may develop distant metastases, avoiding unnecessary surgical treatment and waste of resources. One limitation of our study was the absence of patient survival data, which is currently being collected and will be analyzed in future studies, which currently require a large number of prospective studies to validate.

The best visualization is presented through simple statistical analysis by building a nomogram model. This model calculates a total score based on the values of individual predictor variables and uses the total score to infer the probability of a positive clinical event. It has been widely used in clinical practice in recent years (24), and it is proven to be effective.

In this study, tumor size (P=0.023), low levels of albumin (P=0.024), lymphocyte to monocyte ratio (P=0.044) were independent risk factors for LNM, with age (P=0.072) and white blood cells (P=0.055) (WBCs) slightly greater than 0.05, probably due to the small sample size in this experiment. It has also been previously demonstrated that younger age and higher WBC values are strongly correlated with the spread of tumor cells (25–30)

In our study, LNM was found to be significantly correlated with tumor size (p=0.023), which is consistent with the findings of most scholars (31–33). Previous findings have also focused on the correlation between LMN and tumor size, and although lymph node metastasis was also present in tumors smaller than 1 cm.In general,it appears that larger tumor volumes are more prone to LNM. Larger tumors are capable of directly invading the surrounding lymph nodes by invading the surrounding tissues besides metastasis through the lymphatic vessels since the pancreas lacks a complete envelope. We consider tumor volume to predict the probability of positive lymph nodes, which will help us to adopt an appropriate treatment plan. For smaller tumors, limited resection can be performed laparoscopically to avoid excessive lymph node dissection and damage to surrounding tissues and to improve the prognosis of the patient.

We also analyzed the correlation between the patient’s serum in terms of total protein, albumin and other laboratory indicators and tumor development, and finally found that low protein levels may facilitate the growth and metastasis of tumor cells, which may ne beneficial to predict the probability of LNM (34). In a retrospective study of 207 cancer patients, Adam et al. (24) found that positive lymph nodes are significantly correlated with low albumin levels, and it was concluded that cancer patients are usually accompanied by hypoproteinemia and the subsequent production of ascites and tissue oedema may cause migration of tumor cells, leading to the development of LNM. A related discussion has been found in other studies (35, 36). Alici et al. (37) have suggested that low preoperative serum albumin levels can indicate tumor malignant potential.

A higher probability of LNM occurrence was found in this study with a low lymphocyte to monocyte ratio (<8.152). The possible reason for this result is that monocytes secrete various pro-inflammatory cytokines that promote tumorigenesis, angiogenesis, and distant metastasis, whereas low lymphocyte levels are correlated with poorer tumor control (38). Macrophages are derived from monocytes, and considerable research (39, 40) has suggested that the presence of macrophages may facilitate the growth and migration of tumor cells, which may contribute to the promotion of LNM when the ratio of monocytes is high. Jeffrey W (40) has confirmed through clinical and experimental research that macrophages facilitate the progression of tumor cells. It is influenced by the tumor microenvironment and has a role in promoting angiogenesis, stromal breakdown, and cell motility, as well as producing various mutagenic oxygen and nitrogen radicals and angiogenic factors.

Several previous lineage table studies on the prediction of LNM in malignancy have shown that low age and high WBC counts are also potential independent risk factors. A considerable amount of research (41, 42) has suggested that inflammation is involved in tumor metastasis by altering the immune system status and local microenvironment, and that more WBCs are correlated with carcinogenesis, tumor progression and mortality. In this study, age and WBCs were found to be correlated with LNM with p-values of 0.072 and 0.055, respectively, slightly greater than 0.05.The possible reason for this result is that this study is a systematic review with a small single-center sample size, or possible bias in data collection and processing. However, it seems to be consistent with most scholars’ views.

A review of the literature showed that CA199 is relevant for the diagnosis of early pancreatic cancer, and this has been verified in most studies (43) We suggest that CA199 may also be correlated with the development of LNM, In this study, however, no positive results were obtained. It has also been verified that higher CA199 is a risk factor for lymph node metastasis in early gastric cancer (35). Hopefully, larger medical centers will be able to conduct large sample, multicenter prospective studies to further validate the correlation between CA199 and LNM in pancreatic cancer.

This study also has the above drawbacks (e.g., the small sample size). Because all the information was collected retrospectively, there may have been errors and biases throughout the process. Second, there is sometimes randomness in the removal of peripancreatic lymph nodes when taking pathological tissue, which may result in a lower number of positive lymph nodes in the end than in reality. The 8^th^ edition of the AJCC manual and the College of American Pathologists (CAP) protocol have recommended a minimum number of LNs (eln) of 12 examinations (44). The International Study Group on Pancreatic Surgery (ISGPS) recommends a minimum number of eln of 15 (45). The number of tissue lymph nodes obtained does not meet the above targets. Whereas this last study is a single-center retrospective analysis from northeastern China, further large-sample, multicenter studies and external validation are required to confirm the views of this study.

## Conclusion

A line graph model was established based on the above indicators to predict the probability of LNM in pancreatic cancer. The model has some potential value and takes on a clinical significance in individualized clinical treatment. For patients at high risk of LNM, whether surgical resection and lymph node dissection are appropriate should be considered, and there is some guidance for the choice of radiotherapy.

## Data availability statement

The original contributions presented in the study are included in the article/**Supplementary Material**. Further inquiries can be directed to the corresponding author.

## Ethics statement

The study followed the Declaration of Helsinki. Because of the retrospective nature of the study, patient consent for inclusion was waived.

## Author contributions

XG and BJ designed the research. XL, YL, YX, CX collected, analyzed, and interpreted the clinical data. XG and XS wrote and revised the manuscript. BJ revised the manuscript. All authors contributed to the article and approved the submitted version.
